# Intangible Assets and Performance in Nonprofit Organizations:A Systematic Literature Review

**DOI:** 10.3389/fpsyg.2020.00729

**Published:** 2020-05-05

**Authors:** Ilaria Buonomo, Paula Benevene, Barbara Barbieri, Michela Cortini

**Affiliations:** ^1^Department of Human Sciences, Libera Università Maria SS. Assunta University, Rome, Italy; ^2^Department of Political and Social Sciences, University of Cagliari, Cagliari, Italy; ^3^Department of Psychological Sciences, Health and Territory, G. d’Annunzio University of Chieti and Pescara, Chieti, Italy

**Keywords:** intangible assets, volunteers, performance, intellectual capital, NPOs characteristics

## Abstract

Nonprofit organizations (NPOs) promote citizens’ participation in community life through several different kinds of organizations: some more informal (such as associations and volunteering groups), others more formal or public (such as charities and foundations). This heterogeneity, as well as the well-known peculiarities of NPOs when compared to profit and public ones, poses new challenges to their management. In the constant need to find balance between financial constraints and social value, a main resource for NPOs is the management of intangible assets, such as knowledge, positive relationships within the organization and with users, external image, loyalty and commitment, and so on. From the literature on for-profit organizations, it is well known that proper management of intangible assets improves an organization’s sustainable competitive advantage, not only by enhancing its members’ affiliation and commitment but even by enhancing their productivity. This is particularly relevant when taking into account the main role of volunteers in the third sector. Volunteers, indeed, show different job attitudes and organizational behaviors than paid employees, as their membership and accountability are less formalized and they frequently lack a proper teamwork, due to the high volunteer turnover. At the same time, from the managers point of view, managing volunteers and paid workers require higher skills and competencies than managing human resources in for-profit organizations. Developing these reflections and considerations, we aim to conduct a systematic literature review on the association between intangible assets and performance in NPOs. The literature will be conducted following the indications from the Preferred Reporting Items for Systematic Reviews and Meta-Analyses (PRISMA) statement. It provides an evidence-based minimum set of items to be included in the review, as well as a workflow to properly manage and choose the papers to be included. The authors conducted the research using EBSCO, ProQuest, and Scopus databases.

## Introduction

The literature on the management of for-profit organizations has proven a connection between intangible assets, performance, and innovation potential in these organizations (Romer et al., 1986; Nonaka and Takeuchi, 1995). Intangible assets, such as customer relationships, goodwill, brand recognition, and employee skills, add value to an organization by implementing strategies that respond to market opportunities through developing their internal resources (Sveiby, 1997; Marr et al., 2004). Thus, it is currently well-known that for-profit organizational growth is mainly due to technological advancement, training opportunities, research, and development activities, which, in turn, influence the quality of human, structural, and relational factors within the organization (Bontis et al., 2007).

Several studies have shown that the Intellectual Capital (IC) framework is the primary theoretical approach for the study of intangible assets in organizations. IC allows researchers and practitioners to identify, organize, and break down intangible assets into meaningful dimensions (Young, 2012). This classification, in turn, allows for better management of internal and external resources in organizations, in order to reach objectives and accomplish their mission (Benevene et al., 2019). This paradigm invites practitioners and managers to think about organizational knowledge as expressed within three dimensions: human, relational, and structural/organizational capital. Human capital refers to the knowledge constructed and shared within an organization and includes attitudes, competencies, experience, and skills that are reversed into the organization by the employees (Choo and Bontis, 2002). Thus it is a form of tacit knowledge, unless the organization provides processes and structures to help members foster a valuable use of subjective experiences and resources (Ordonez De Pablos, 2004). Several authors claim that human capital has the most significant impact on the generation of intangible organizational resources when compared to other IC dimensions, as it influences the acknowledgment and the implementation of other organizational resources (Guest et al., 2003; Wright et al., 2003; Paauwe and Boselie, 2005; Paauwe, 2009; Jiang et al., 2012; Manuti and de Palma, 2018; Manuti and Giancaspro, 2019). Relational capital is about relationships with all the stakeholders, as well as an organization’s external image. Furthermore, relational capital demonstrates how knowledge is shared and negotiated with external actors (i.e., partners, competitors, users, suppliers) (Sveiby, 2001; Grasenick and Low, 2004). Finally, structural/organizational capital includes the processes, models, routines, leadership styles, organizational culture, and patents aimed at influencing and supporting human capital (Sveiby, 2001). A recent literature review highlighted that employees’ knowledge, structural and organizational arrangements, and valuable relations support each other in enhancing both performance and innovation in for-profit organizations (Inkinen, 2015). This connection is particularly fruitful when IC informs human resource management (Yang and Lin, 2009; Wang and Chen, 2013).

More recently, intangible assets and IC have emerged as relevant also for nonprofit organizations (NPOs). NPOs are private, independent, self-governed organizations, based on voluntary participation, whose profit is not distributed to individuals or owners, but reinvested in the organizational mission, that represents a contribution to the public good or the general welfare (Salamon and Anheier, 1992). NPOs, indeed, are aimed at creating social value and maximizing social utility, while not considering financial profit as their main objective (Bahmani et al., 2012). In other words, NPOs deliver services that are intangible and rely on intangible assets, such as volunteers’ loyalty, a good name, and relationships with other organizations (Benevene and Cortini, 2010; Kong and Ramia, 2010; Dal Corso et al., 2019). Thus, IC management responds to NPOs’ peculiarities as it maximizes their intangible resources, allowing for a more visible provision of services to the community (Kong, 2003). The lack of focus on profit maximization, as well as the mission of NPOs, pose new challenges to their management. This is particularly true when considering the growing number of requests that society makes to these organizations in terms of fulfilling social needs, providing services, and protecting environmental and cultural contexts. In other words, NPOs are now called to fulfill basic community needs, while receiving fewer public funds and competing between each other to have more donors (Bahmani et al., 2012). IC and its management emerges as a valuable paradigm for NPOs, as these organizations are “knowledge intensive,” meaning that the achievement of their objectives depends on the human resources brought by staff and volunteers, more than by tangible, physical capital (Hume and Hume, 2008; Kong, 2010). The crucial point is then to understand how to develop new resources from the existing ones, which is precisely why effective management of IC is pivotal.

### Intellectual Capital in NPOs

Many studies have shown that NPOs would benefit from proper IC management. Kong and Prior (2008) proposed a conceptual framework based on the IC paradigm for NPOs. The framework states that the three IC dimensions offer a potential avenue for competitive advantage, as long as they are interrelated and fulfill organizational, donor, and user needs. The tool required to reach the sustainable advantage is a strategic IC management that would allow an adequate control over the knowledge flows within and outside the NPO, as well as the value creation emerging from these flows. According to the authors, proper recognition of how knowledge is generated and vehiculated internally and externally would help managers to make better strategic decisions for the NPO (Kong and Prior, 2008). A valuable example regards the volunteer workforce. Volunteers, indeed, show different job attitudes and organizational behaviors than paid employees, as their membership and accountability are less formalized, and they frequently lack proper teamwork due to the high volunteer turnover (Skoglund, 2006; Hustinx, 2010). At the same time, from the managers’ point of view, managing volunteers and paid workers requires higher skills and competencies than managing human resources in for-profit organizations (Barbieri et al., 2018; Benevene et al., 2018). The few studies available on NPOs highlight that volunteers benefit from a team and cooperation-centered leading approach, showing higher engagement and higher satisfaction when their managers strengthen these aspects of their work (Dal Corso et al., 2019). Engagement and satisfaction, in turn, are likely to improve their performance. In other words, proper strategic management of human resources in NPOs requires managers to take into account intangible assets (Lettieri et al., 2004; Clarke et al., 2011). At the same time, other authors claim that knowledge management in NPOs has neither the same objectives nor the same implications as it does in for-profit organizations. First of all, knowledge management in NPOs is not aimed at increasing financial gain tout court, as is the case in for-profits, but at sustaining organizational mission and aims (Kong, 2007; Sillanpää et al., 2010; Bloice and Burnett, 2016). Secondly, barriers that could impede knowledge sharing in for-profits and NPOs are different. Starting from Riege’s (2005) list of barriers to knowledge generation in business firms, Bloice and Burnett (2016) added several barriers for NPOs, related to the peculiar missions and structure of these contexts. Riege’s list, indeed, individuated three types of barriers: individual (e.g., infrequent or ineffective interactions among employees), organizational (e.g., poor culture or structure), and technological (e.g., reluctance to introduce innovations). Bloice and Burnett (2016) claimed that some typical characteristics of NPOs, such as the high volunteer turnover, the lack of strategic planning, and the inherent competition between the altruistic organizational mission and the competitive objectives (Kong, 2007, 2010; Hume and Hume, 2008; Ragsdell, 2009, 2013) act as barriers to knowledge sharing. This point is consistent with findings focusing on the lack of awareness about several aspects of IC in NPOs. Some authors, indeed, showed that managers of NPOs tend to perceive human capital as the most important dimension, while undervaluing structural/organizational capital (Curado, 2008; Kong, 2008). This approach may threaten the knowledge management system, by neglecting the role of organizational routines and processes (Benevene et al., 2017).

Previous literature on the subject has analyzed the impact of IC and knowledge management system in NPOs and provides some hints about the potential impact of these intangible dimensions on NPOs’ performance. At the same time, current literature still needs a systematic analysis of whether and how volunteer groups, foundations, charities, and associations deal with this management, and what are the results of this process, in terms of their performance and overall impact on society. Effective management of IC requires the assessment of both individual and organizational performance.

### Performance in NPOs

In the managerial literature, organizational performance is determined by the results of an organization when compared to its goals and objectives (Cho and Dansereau, 2010; Tomal and Jones, 2015). Organizational performance may be measured with objective or subjective criteria (Andrews et al., 2006). Objective criteria include impartial and independent indicators that could be externally verified in their accuracy (e.g., number of users in a social care service). Subjective criteria, on the contrary, refer to internal informants (e.g., managers), or to external informants whose judgment cannot be scrutinized (e.g., customers measuring their satisfaction toward the firm). Both criteria, to be valid, need to focus on meaningful performance dimensions according to organizational aims and scopes. For this reason, despite the common notion, according to which objective criteria are more valid than subjective ones, objective indicators may not reflect the actual nature and complexity of the assessed organization, tackling more legal and technical requirements than goal achievement (Andrews et al., 2006), while subjective measures may be better situated in the assessed context (Richard et al., 2009). Thus, the choice of performance measures may depend on several considerations, from the scope of the assessment to the nature of the organization (Richard et al., 2009).

As far as the NPOs are concerned, the operationalization and measurement of performance within the third sector pose a further challenge for scholars and practitioners. A first issue regards the absence of a financial bottom line that could act as an evaluation criterion (Kong, 2010). Another point regards the multiplicity of stakeholders that an NPO is required to deal with: from the government to its donors and funding organizations, and from users to volunteers (Moxham, 2014). Accordingly, Moxham (2009) reported four reasons why NPOs are urged to measure their performance (financial reporting, demonstration of achievements to external market actors, users, and members, operational control and support to innovation), showing that different requirements come from different stakeholders and produce different performance measurements. According to the literature on for-profit organizations, indeed, performance measurement in NPOs are differentiated between internal and external, objective and perceptual/subjective (Sowa et al., 2004), and efficiency and effectiveness-related performance (LeRoux and Wright, 2010). At the same time, financial and mission-based performance measures can be distinguished (McDonald, 2007; Bontis et al., 2018). Financial performances regard how NPOs acquire and use their funding and rely on laws about NPOs’ economic accountability (Moxham, 2009, 2014). Mission-based performances include the activities pursued by the NPOs in order to have a social impact: for this reason, some common indicators are the number of volunteers, employees and users, the services provided, and the satisfaction perceived by users and staff (Epstein and McFarlan, 2011; Andreaus and Costa, 2014). Generally speaking, volunteer and employee workload, user numbers, customer satisfaction, staff meaning, and external audits are mentioned in literature as the most frequently used in NPOs (Sowa et al., 2004; LeRoux and Wright, 2010; Álvarez-González et al., 2017). Moxham (2014) showed that the key driver for performance measurement in NPOs is financial accountability toward a public funder. This point has a double implication. First, it raises questions about the adaptability of public criteria for financial performance evaluation for NPOs. Second, it sheds light on the low strategic use of performance assessment in NPOs. Similar to the use of IC management, as long as the performance assessment is not implemented in the context of aware, fruitful strategic planning, NPOs are at risk of losing crucial resources.

### Objectives

Building on these considerations, the authors of this study aim to conduct a systematic literature review on the association between intangible assets and performance in NPOs. Preliminary searches were done to determine that there is no published review addressing this question in the last 10 years.

## Methods

The literature review has been conducted following the guidelines from the Preferred Reporting Items for Systematic Reviews and Meta-Analyses (PRISMA) statement (Moher et al., 2009). The eligibility criteria regarded empirical studies published in peer-reviewed full-length articles, from 2005 to 2020, written in English. The period of literary research lasted from December 2019 to February 2020.

Due to the small number of studies dealing with this subject, no further criteria for exclusion are used. Even though this choice does not allow for further quality checks (except the manual exclusion after abstracts reading), given the decision to include only peer-reviewed papers, it seems reasonable to not exclude other works.

### Information Sources and Search Strategy

The following databases and search engines were employed for the search: EBSCOhost, ProQuest, and Web of Science. Several terms were used to implement the search strategy. Each database required a different detailed strategy. At the same time, the following generic strategy covers the focus of our research:

(intangibles or “intangible assets” or “balance scorecard” or knowledge) AND (nonprofit or nonprofit or “not for profit” or voluntary or “third sector” or NPO or NPOs) AND (performance or outcome or effectiveness or achievement or productivity)

Considering the overlap between theoretical considerations on intangible assets and the IC framework, in the second round of search, on the same databases and in the same time range, the authors chose to add keywords that would allow the identification of papers addressing intangible assets, with an IC framework approach. For this reason, the authors’ final search strategy was the following:

(intangibles or “intangible assets” or “balance scorecard” or knowledge or “intellectual capital” or “human capital” or “relational capital” or “structural capital” or “organizational capital”) AND (nonprofit or nonprofit or “not for profit” or voluntary or “third sector” or NPO or NPOs) AND (performance or outcome or effectiveness or achievement or productivity)

According to the needs, the keywords were searched in the publication title or abstract.

### Data Collection Process

All references were gathered in a Mendeley database, and duplicate references were removed. Two authors independently reviewed the chosen references, deciding to exclude further papers eventually. Papers were analyzed with respect to their content, and papers with content that were not fully within the scope of this review, due to knowledge transfer processes or best practices in NPOs and not within empirical research, were eliminated. In the reference scrutinization phase, the authors looked for papers written in the English language that contain keywords of interest in the title, and then scrutinized the papers’ abstract to check the inclusion criteria.

## Results

### Study Selection

Of the sixteen full-text articles assessed for eligibility, seven papers did not address this review regarding the association between IC and performance in NPOs. The remaining nine papers were determined as eligible and were included for review (see Table 1).

**TABLE 1:** Descriptions of papers addressing the association between IC and Performance in NPOs.

References	Country	Study	IC dimension	Performance	Type of	Participants	Participants
		methodology		measure	NPO	(number)	(NPO role)
Misener and Doherty, 2013	Canada	Qualitative	R	Subjective	Sport-related	20	NPOs presidents
Mohd Noor et al., 2015	Malaysia	Quantitative	H, R, S/O	Subjective	Any	271	NPOs employees
Zhu et al., 2016	Canada	Quantitative	H, S/O	Subjective	Any	376 (156 from NPOs)	NPOs directors
Wemmer et al., 2016	Germany	Quantitative	R	Subjective	Sport-related	292	NPOs directors
Cady et al., 2018	United States	Quantitative	H	Objective	Farming	285	NPOs members
Álvarez-González et al., 2017	Spain	Quantitative	R	Subjective	Any	325	NPOs board members
Benevene et al., 2018	Italy	Qualitative	H, R, S/O	Subjective	NPOs socio cooperatives	70	NPOs managers
Bontis et al., 2018	Italy	Quantitative	H, R, S/O	Objective, Subjective	NPOs socio cooperatives	151	NPOs board members
Vakharia et al., 2018	United States	Quantitative	H, R, S/O^*a*^	Objective, Subjective	Arts-related	368	not specified

*H, human capital; R, relational capital; S/O, structural/organizational capital. ^*a*^IC dimensions were reorganized in a new construct.*

### Study Characteristics

The included works revealed themes that could be reorganized as expressions of the IC framework, despite showing peculiarities in their application (Table 2 shows a synthesis of the terms, variables, and dimensions used to measure each dimension). Overall, the three dimensions of IC were equally represented in the selected works. At the same time, the references to the IC framework, as well as to each of the three dimensions, were heterogeneous in the selected papers. More specifically, three out of nine papers clearly stated the intention to measure one or more dimensions in the IC framework (Mohd Noor et al., 2015; Benevene et al., 2018; Bontis et al., 2018). Three out of nine papers addressed all the dimensions of IC: human, relational and structural/organizational (Mohd Noor et al., 2015; Benevene et al., 2018; Bontis et al., 2018). One out of nine papers only addressed the human dimension (Cady et al., 2018), whereas three papers addressed the relational dimension (Misener and Doherty, 2013; Wemmer et al., 2016; Álvarez-González et al., 2017), and one paper addressed both human and structural/organizational capital (Zhu et al., 2016). Finally, one paper took into account dimensions related to the types of knowledge tackled in the IC framework but reorganized them in the knowledge-centricity construct (Vakharia et al., 2018).

**TABLE 2:** Variables related to the IC framework in each paper.

IC dimension	Terms, variables, and dimensions	References
Human capital	Knowledge sharing (perceptions and beliefs)	Mohd Noor et al., 2015
	Board information (availability and use)	Zhu et al., 2016
	Volunteer motivation, Volunteer efficacy	Cady et al., 2018
	Narratives	Benevene et al., 2018
	Narratives	Bontis et al., 2018
	Levels of staff capacity, training, and roles	Vakharia et al., 2018
Relational capital	Interorganizational relationships	Misener and Doherty, 2013
	Beneficiary participation	Mohd Noor et al., 2015
	Interorganizational relationships	Wemmer et al., 2016
	Interorganizational relationships	Álvarez-González et al., 2017
	Narratives	Benevene et al., 2018
	Narratives	Bontis et al., 2018
Structural/organizational capital	Collaborative culture	Mohd Noor et al., 2015
	Board strategic involvement	Zhu et al., 2016
	Narratives	Benevene et al., 2018
	Narratives	Bontis et al., 2018

The performance of NPOs was measured and reported using several measures across the studies. While four studies used a scale on organizational performance or effectiveness, measuring how it was perceived by the NPOs’ members by means of validated or ad hoc Likert scales (Mohd Noor et al., 2015; Wemmer et al., 2016; Zhu et al., 2016; Álvarez-González et al., 2017) or structured interviews (Misener and Doherty, 2013; Benevene et al., 2018), others relied on objective measures of performance. Among the objective measures selected, a number of activities, members, and beneficiaries were used as performance measures in two studies (Álvarez-González et al., 2017; Cady et al., 2018), as well as financial performance (Bontis et al., 2018; Vakharia et al., 2018). Furthermore, one study considered mission-based as well as financial performance (Bontis et al., 2018).

With regard to the types of NPOs involved, participants in most studies (five out of nine) were gathered independently from the kind of NPOs they belonged to (Mohd Noor et al., 2015; Zhu et al., 2016; Álvarez-González et al., 2017; Benevene et al., 2018; Bontis et al., 2018). Two studies focused on sports-related NPOs (Misener and Doherty, 2013; Wemmer et al., 2016), one on a farmers’ association (Cady et al., 2018), and one on a performing arts association (Vakharia et al., 2018).

### Synthesis of Results

Overall, the selected studies showed that IC dimensions are related or have an impact on NPOs’ performance. Studies addressing two or all the dimensions of IC in the same model showed differentiated effects on performance. Within these models, human capital and relational capital are strongly related with NPOs performance, above all in terms of internal and external effectiveness (Mohd Noor et al., 2015), perceived quality of work (Benevene et al., 2018), and financial and mission-based outcomes (Benevene et al., 2018; Bontis et al., 2018). At the same time, structural/organizational capital has a lower impact on performance measures in the above-mentioned studies. Indeed, it is reported as showing null effects in the papers published by Mohd Noor et al. (2015) and Bontis et al. (2018), both quantitative studies, and as having an impact on the quality of work in the qualitative work by Benevene et al. (2018) and in the model implemented by Zhu et al. (2016). Studies tackling one dimension from the IC framework addressed human or relational capital. Cady et al. (2018) measured human capital as NPOs members’ self-efficacy, collective efficacy, and organizational support, showing its effect on NPOs’ financial performance through the effort devoted to the tasks. Relational capital was mainly studied within sports-related NPOs (Misener and Doherty, 2013; Wemmer et al., 2016), and operationalized as cooperation with competitors (coopetition, Wemmer et al., 2016). According to these studies, cooperating with other NPOs with a similar mission provides access to higher financial, physical, and human resources, thus promoting innovation and, consequently, better performance through the use of external knowledge. The third study addressing relational capital (Álvarez-González et al., 2017) looked at NPOs more broadly and showed that competition among NPOs and business firms improves the effectiveness of the organization, in terms of obtained funding (financial performance), provided services, and reached users (mission-based performance). Finally, a study reorganized the dimensions of IC into a new construct, namely the knowledge centricity (Vakharia et al., 2018). Knowledge centricity includes a hard and a soft dimension. The hard dimension includes elements related to human capital (staff abilities related to data collecting and managing, technology, and reporting, the level of staff capacity, training, and roles). The soft dimension includes elements related to structural/organizational capital (level of board engagement; strategic use of audience data for programming and audience development). The authors showed that both dimensions have a role in enhancing financial resilience (namely the months of available capital and cash) in NPOs with a mission in the performing arts industry.

## Discussion

Overall, the authors found few studies about the association between intangible assets and organizational performance in NPOs. As Table 2 highlights, the most part of intangible assets mentioned in the selected studies could be reconducted to the IC theoretical framework. At the same time, previous literature tackling IC and organizational performance inform us about the cruciality of these dimensions for NPOs to grow and be competitive (Kong, 2003; Moxham, 2009, 2014; Benevene and Cortini, 2010). Building on studies that addressed the perceived importance of IC and performance measurement in the third sector (Moxham, 2009; Benevene et al., 2019), it is apparent that most IC management and performance evaluation is conducted in an informal, non-planned way. By relocating NPOs’ informal knowledge management process in the IC framework, the authors aim to raise the awareness of managers and practitioners on these topics, while moving from informal knowledge management to an effective, strategic one.

### Summary of Evidence

All the studies selected in the literature review showed an effect of one or more dimensions of IC on NPOs’ performance. While the previous section described the results of the studies according to the number of dimensions tackled in the study and the type of study conducted, the following section will reorganize and comment on the results considering the three dimensions of IC. According to the organization detailed in Tables 1 and 2, studies that considered members’ knowledge (beliefs and perception about the organization, their roles or their mansions) were classified as tackling human capital. Studies that considered the relationships between NPOs and other organizations were classified as tackling relational capital. Finally, studies considering aspects of an NPO’s structure, routines, or culture were classified as tackling structural/organizational capital. As described above, the same study could be considered as tackling one or more IC dimensions.

With regard to human capital, the knowledge shared by the employees and volunteers in terms of education, training, and procedures, as well as their motivation, sense of efficacy, and perceived support emerge as the most important elements for NPOs to succeed. Interestingly, this association is confirmed in all the studies, independently from the performance measure used. This point is consistent with the idea that in NPOs, “people are the most decisive factor” (Benevene et al., 2019; p. 21). Human resource management in NPOs, indeed, would allow managers to better tackle NPOs’ peculiarities by, for example, strategically planning the mission and the combination of paid and non-paid workers and by providing differentiated motivational structures for employees and volunteers (von Eckardstein and Brandl, 2004). By planning and intervening on age structures, strategic addressing of staff management in NPOs would lead to on balance higher performance quality between paid and non-paid employees in the requirements and qualifications necessary to be included in the staff, coordination and leadership, remuneration, training, and development of human resources (von Eckardstein and Brandl, 2004). The centrality of employees and volunteers in delivering services is consistent with the literature on the impact of a people-based approach in human resource management on organizational success and performance. According to this approach, employee performance is not simply related to individual perceptions about HR management strategies, but also to the acknowledgment of the experiences and perceptions of coworkers (Kehoe and Wright, 2013; Posthuma et al., 2013; Saks and Gruman, 2014). Such findings on team management and its implications for individual commitment and outcomes reinforce even more the idea that strategic management of teams would be highly beneficial for NPOs, especially when considering the high heterogeneity of the teams that work for NPOs (e.g., mixed employees-volunteers’ teams).

Regarding relational capital, one of its most studied dimensions is NPO-organization cooperation (Misener and Doherty, 2013; Wemmer et al., 2016; Álvarez-González et al., 2017). Described as the relationship between NPOs or with public organizations or business firms, it seems crucial for NPOs to have partners that share missions or users to achieve better resources, higher success, or formal advantages (e.g., accreditations). Interestingly, apart from the studies mentioned in this review with regard to the impact of partnerships on performance, most of the studies on NPO collaborations regard public-nonprofit partnerships (Gazley, 2017). Government nonprofit collaborations, indeed, are abundant in the contemporary policy environment (Peng et al., 2020). According to Peng et al. (2020), NPOs are likely to maintain their collaboration with public organizations for two forms of commitment: continuance commitment, mainly due to the governmental funds and their importance for the future of NPOs, and affective commitment, related to the identification of the NPO in the collaboration, as well as to the importance given to the partnership for the internal and external image of the NPO. Despite this still not having been verified in the literature, these forms of commitment are likely to have an impact on the effectiveness and the quality of work in NPOs, as already found in for-profits (e.g., Bloemer et al., 2013). A second aspect mentioned in the literature addressing the impact of external relations on NPOs’ performance regards the NPO-users relationship. This relationship is considered as beneficiary participation, namely the likelihood that users influence and share control over NPOs initiatives and resources (Mohd Noor et al., 2015), and is also considered as general importance given by the NPOs to its stakeholders, because most of the physical, financial, and human resources depend on the reliability and accountability communicated toward the external environment (Benevene et al., 2018). Both the relationships (NPO-organization and NPO-users) have been significantly associated with performance in all studies addressing this IC dimension, independently from the performance measure used. Another interesting point of view, still not addressed in the IC-performance literature, comes from the organizational image framework. Organizational image, indeed, is commonly described as a perceptual internal image and a construed external image that is respectively based on employees/volunteers’ perception and outsiders’ judgments (Rho et al., 2015). According to Rho et al. (2015), the construction of organizational image both within the NPO and toward the external context has an impact on employee and volunteers’ intention to leave or commit to the NPO by means of the identification process. Again, it is likely that the higher the commitment and the lower the intention of an employee or volunteer to leave an organization, the higher the quality of work, and consequently, the overall quality of organizational performance.

Structural/organizational capital seems to be less studied and has less implicated dimensions when considering the association between intangible assets and NPOs’ performance. It is interesting to note that the role of structural/organizational capital strongly emerges in the qualitative study included in the review (Benevene et al., 2018), and in the study on strategic board involvement (Zhu et al., 2016), as a valuable dimension for organizational performance. This may be due to the variety of measures used to tackle the heterogeneity of structural/organizational capital, which was measured as a combination of organizational information about the NPOs (including the services provided, the kind of users, the certifications (Bontis et al., 2018), as collaborative culture (Mohd Noor et al., 2015), and as strategic board involvement (Zhu et al., 2016), depending on the considered studies. The lack of consensus on the measurement of structural/organizational capital across the selected studies may have had an impact on the scarce implications for the performance of NPOs. This is particularly true when comparing the variables used in the mentioned studies with the description of structural/organizational capital that emerged in structured interviews. Benevene et al. (2018), indeed, reported that senior managers from small and medium nonprofit socio-cooperatives individuate among the key indicators of structural/organizational capital the absence of bureaucracy, the centrality of employees, and the horizontal organizational structure. Despite low or no overlapping among the variables measured by quantitative studies and the NPOs managers’ descriptions, it is likely that the participants’ construction and communication of their meanings permit emergence of structural/organizational capital’s role in a NPO’s effectiveness. Furthermore, several authors claimed the importance of considering all the dimensions of IC when managing knowledge in NPOs (Kong, 2003). Consistently, frameworks addressing NPOs management underline the necessity of strategically planning the organization of work in the third sector, with regard to the definition of tasks and their context of accomplishment (individual vs. team-based), as well as to the structural relationships among paid employees and volunteers in cooperating toward the accomplishment of organizational goals (von Eckardstein and Brandl, 2004).

### Limitations

First of all, the literature review was built on a small number of papers, despite representing all the works in the field addressing our research question. At the same time, the authors believe that the low number of works is not due to the low importance of this topic for NPOs’ performance, but to the general tendency to consider NPOs as informal organizations (Benevene et al., 2017). Secondly, most of the papers regard participants from Western countries, thus defining a gap concerning whether and how the mentioned results remain valid for non-Western countries.

## Conclusion

This review showed that the literature on intangible assets and performance is mainly related to the IC framework, highlighting that, despite the different impacts and implications, all of the IC dimensions have a role in influencing performance. The low attention given to the structural dimension of IC reflects the lack of attention of NPOs toward formal strategic planning. Despite this, our results suggest that a more IC focused planning and management process would lead to higher quality and effectiveness in NPOs’ performance. Hopefully, the first definition of the impact of IC on NPOs’ performance would inform and encourage researchers and practitioners in tackling more of these topics when addressing and intervening in the third sector. At the same time, the authors’ results appear to be the first in the literature to indicate the importance of further analyzing the role of IC on NPOs due to the emergence of several indicators of IC as potential predictors for organizational performance. Further research could shed new light on the role of those factors on the quality of work in NPOs, by verifying whether patterns of influence already found in for-profits could be confirmed in NPOs. Finally, the fragmentation in the measurement methods and tools, above all when addressing organizational performance, calls for a better classification of performance measures in NPOs.

## Author Contributions

All authors, IB, PB, BB, and MC developed the research project and reviewed the literature. IB carried out the data analysis.

## Conflict of Interest

The authors declare that the research was conducted in the absence of any commercial or financial relationships that could be construed as a potential conflict of interest.
